# The integration of occupational- and household-based chronic stress among South African women employed as public hospital nurses

**DOI:** 10.1371/journal.pone.0231693

**Published:** 2020-05-01

**Authors:** Jennifer Cohen, Willem Daniel Francois Venter

**Affiliations:** 1 Department of Global and Intercultural Studies, Miami University, Oxford, Ohio, United States of America; 2 Ezintsha, Department of Medicine, Faculty of Health Sciences, Wits Reproductive Health and HIV Institute, University of the Witwatersrand, Johannesburg, South Africa; Paris School of Economics, FRANCE

## Abstract

**Background:**

Nurses are a critical part of healthcare delivery systems, especially in under-resourced environments. Compared to other female-dominated professions in South Africa, nurses are securely employed and relatively well-paid. However, they are often drawn from complex, poor communities where they are responsible for many dependents and must accommodate community and family expectations of financial, health, and other forms of support.

**Aim:**

The aim of the study was to explore public hospital-employed, black women nurses’ lived experiences to better understand their stressors and consider interventions that may reduce psychological distress.

**Methods:**

In 2015, we conducted semi-structured life history interviews with 71 nurses in Johannesburg. Using grounded theory and social network mapping, we trace complex, interrelated stressors and networks of familial dependency.

**Results:**

Every participant experienced high levels of stress. Nurses described daily lives of chronic distress, with extreme pressures on their incomes, time, and resources. Much of the pressure on nurses comes from familial and partner dependency, both in absolute number of dependents and intensity, and related financial obligations and debt. Dependency is a function of social and cultural norms which assign women primary responsibility for unpaid work, yet nurses characterized their efforts as unsustainable and anxiety-inducing; their pay and paid work schedules made meeting that responsibility virtually impossible.

**Conclusions:**

The structure of the nursing occupation contributes to stress outside the workplace, while the structure of nurses’ households contributes to stress and emotional exhaustion. The integrated nature of their chronic stress suggests that occupationally-oriented interventions are unlikely to adequately address it. To fully alleviate chronic stress, the gender norms that place responsibility for unpaid work on nurses with already full-time employment need to shift. A better understanding of the extensive networks dependent on nurses should inform interventions designed to improve their wellbeing. Assistance addressing childcare, mental health, and financial planning may be especially useful.

## Introduction

*I’m just trying to keep things intact*. *I’m just trying to survive between the two worlds*. *I think a lot about it*. *At times I don’t even want to think about it either*. *I don’t want to focus a lot on that because*, *eish*, *I know it can really mess me up…The pressure can push me to an extreme that I don’t want to go to*.

—A staff nurse (R1I15) and single mother, previously diagnosed with depression, describing coping with nursing and life outside of nursing

There are far too few nurses for the health needs of the South African population, with training taking substantial time and resources [1]. In many areas, nurses are the most senior healthcare providers available, making their continued effective participation in the health system of paramount importance to public health. However, little is known about their lives or how the structures of their households and workplaces contribute to chronic stress and burnout.

In South Africa nursing offers secure employment and relatively high pay, but is notoriously stressful. Nurses’ shift schedule, moonlighting for extra income (often illegally), their sometimes-uneasy relationships with doctors and other health cadres, violence in the workplace from families and patients, contact with infectious diseases, exposure to death and ill patients, and the intimate human services nature of the work presumably contribute to stress. Nurses are also expected to conform to gender roles around housework, childcare, eldercare, and care for ill or disabled family members, with very limited childcare facilities available for those doing shift work. Nurses, predominantly black women in South African context, are often drawn from poor communities with high unemployment rates.

Four out of ten black^i^ workers are unemployed, therefore demands are commonly placed on the employed by immediate and extended family members [2].

^i^ The apartheid government’s racist terminology distinguished between four populations groups: “African,” “Coloured,” “White,” and “Indian.” Although the term “black” is sometimes used to identify all peoples oppressed under apartheid, in this article we use the term “black” primarily to mean African. The 71 participants in the study, 70 of whom were “Africans” and one of whom was “Coloured.”

A paid worker may provide critical financial support to upwards of 10 or more family members [3].

Historically, the sole professional occupations available to educated black women during apartheid were nursing and teaching, and later, social work and police-work [4]. Professional status positioned nurses, especially registered nurses, at the “top of the social structure in the township community” [5]. Pay for nursing was low overall, but black nurses earned far more money than those in the few remaining occupations available to black women, mainly domestic work or self-employment. For much of the twentieth century, nursing was considered one of the, if not *the*, most prestigious professions for black women [6].

Nurses were sought after wives both during and after apartheid, thought to be sophisticated and dignified and as having learned qualities needed in a wife and mother [4,6]. An interviewee in another study notes, “They say we are jackpots” [7]. With secure jobs and earnings above the median for Africans, many nurses are the main source of income in their households.

For nurses living with a partner, conventional intra-household bargaining models predict that nurses’ salaries and job security increase their bargaining power in household negotiations [8]. Nursing offers stability in the event that negotiations within the household break down, for example in the case of divorce, and enables some nurses to form households without partners to begin with. However, within households, income alone may not trigger transformative change in power dynamics, and negotiations take place in specific contexts where other parameters also matter [9–11]. In a context in which women largely, “accept the authority of their husband and…assume sole responsibility for the household and child care…,” the space for negotiating is limited [12]. Men are not expected to, and typically do not, engage in the reproductive labor assigned to women. Childcare is the responsibility of women alone, whether or not a couple is married or living together [13]. The operation of these gender roles in a racialized environment of high unemployment, low pay, high mortality/morbidity, and extreme inequality put a unique burden on black women. Between paid work and work in the home, nurses’ workload can contribute to chronic stress.

In the past 15 years, nursing has been overshadowed by better paying, more attractive career opportunities for black women [14]. Nurses’ health and the nursing occupation are pressing issues in public health. Despite rapid growth in nursing in the 20^th^ century, there is a persistent shortage of nurses–data is suggestive of a shortage on the order of between 35 and 45%–in the public sector, related to slower growth in new registrants, as well as retirements and resignations [14,15]. Causes of attrition include disillusionment with the profession, emigration, HIV/AIDS among nurses, reduced occupational status of nurses relative to historical status, and chronic stress.

### Motivation

Nurses are critically important in domestic and global public health, and the global supply of nurses falls short of demand. This study of nurses in South Africa pays close attention to the local context in which nurses work. Still, some of the findings may be of use for understanding chronic stress, absenteeism, and burnout among nurses elsewhere, particularly where gender norms dictate that women bear responsibility for household work and childcare.

Nursing is dominated by black African women, the largest demographic group in South Africa (41.4%) and a population likely to support dependents [16,17]. In a previous interview-based study in Johannesburg the principal investigator (PI, the first author) found that higher familial dependency and fewer incomes in women’s households contributed to women and men street traders’ different subjective experiences of paid work, which women reported was less secure despite their nearly identical earnings [18]. Studying securely employed nurses permits sharper focus on stressors across different spaces in nurses’ lives, primarily the hospital and the home. Nurses employed in public hospitals are a population of particular interest because their health is of great consequence to the health of the population, the vast majority of which does not have access to private care [15].

There is little data on the prevalence of chronic stress or mental illness among nurses in South Africa; however, given their occupational demands, it is unlikely to be lower than the general population, where undiagnosed anxiety and depression are very high (lifetime prevalence of anxiety disorders 15.8%; mood disorders 9.8%), especially among women and those engaged in shift work [19–21]. The aim of the present study was to explore public hospital-employed female nurses’ lived experiences, including their family, work and financial lives, to better understand their stressors and consider interventions that may reduce psychological distress.

We conducted an intensive interview-based analysis of nurses employed in a large, urban, public teaching hospital in South Africa, using qualitative sampling and interviewing techniques, to establish sources of stress. We used accepted research and data collection methods for this study, which are summarized in the following section. Throughout the paper we follow the Consolidated Criteria for Reporting Qualitative Research (COREQ) per PLOS One guidelines [22].

## Methods

The PI conducted semi-structured life history interviews with 71 nurses employed in a public academic hospital in central Johannesburg, South Africa.

There is precedent of using qualitative methods in nursing going back to the 1960s and of using life history interviewing techniques [23–29]. Life history interviews are a narrative research method that may be used for case studies of professions [30,31]. Life history research can be deeply personal, and topics broached in this study included traumatic events like rape and suicide, as well as happier subjects like seeing patients recover and love of one’s self and family. The study takes a grounded theory approach. We do not interpret grounded theory as a specific method, but as a style of qualitative analysis using interviews, taking seriously the influences of social structures on respondents’ experiences, and generating substantive theory [32,33]. Inductive reasoning is the framework through which details about nurses’ experiences reveal common themes and therefore a more general picture of the worlds nurses inhabit. The study is informed by descriptive, non-essentialist phenomenology, with rich description of the lived experience, and critical ethnography, which highlights power relations among groups [24,29]. This paper focuses on describing systematically-collected data about nurses’ stressors in the context of their households and South African society [23].

### Ethics

The project was approved by the Human Research Ethics Committee at the University of the Witwatersrand [H19/08/05] and the Institutional Review Board [IRB 14/15-61] of Whitman College, the PI’s employer at the time of the study, and relevant senior hospital officials.

### Data collection and sampling

All interviews were completed by the PI, a white female professor (PhD Economics) from the United States, who is trained in, and has extensive experience conducting qualitative research in South Africa. Participants were recruited during in-person interactions in randomly selected hospital wards and day clinics, excluding emergency and trauma due to concerns about disrupting urgent care. The PI introduced herself to potential participants as a professor affiliated with a local university, the University of the Witwatersrand, where she was a Research Associate in the Society, Work, and Development Institute. As an economist who studies gender, work, and health, the PI chose to study public sector nurses because the high-stress occupation is critical to public health; information that was also shared with participants prior to interviews. A document describing the study and the consent process was made available during scheduling of the interviews, and was re-presented to participants at the start of the interviews. Pilot interviews were conducted individually, one with a nursing professor and one with a nursing student.

The interviews took place at the participants´ workplaces in private rooms with only the interviewer and the participant present. Approximately 75% of the nurses approached by the PI chose to participate; those who declined were not asked to explain their reasons given the sensitivity of the topics covered. After gaining written informed consent, detailed data were collected on age, marital status, education, union membership, employment history, family background, time use, household structure, household members’ demographics, details on household networks, indebtedness, sources of debt, monthly income, paycheck deductions, and remittances and other expenditures. The interviews were supported by a questionnaire (S1 Questionnaire) with open-ended questions such as: “What do you like about your work here in the hospital?” Interviews were conducted in English, a language in which all participants were fluent. They were recorded and transcribed by the PI and an assistant, and coded by the PI. Transcripts were not returned to participants for comment but participants were given the PI’s contact information to discuss the project or their interview further if they wished. Themes were identified in the coding process. During interviews field notes and income/expenditure information were recorded by hand or directly into budget spreadsheets. Time-use data were collected from recall.

The study used purposive sampling and the PI employed the concept of saturation to guide the sample size. Saturation calls for conducting as many interviews as produce new themes or codes for answering a research question [34]. Once interviews produce no new themes, interviewing may end. Guest et. al (2006: 60) note that saturation has “become the gold standard by which purposive sample sizes are determined in health science research” [34]. Recommended guidelines for ethnography and grounded theory are between 20 and 36 interviews or six to eight for homogeneous samples and 12 to 20 for heterogeneous samples [34].

The PI conducted in-depth one-time interviews with 71 nurses in 19 wards and clinics. The study reached saturation, in terms of generating new themes, within about 14 interviews per tier of nurses (42 total). In the interest of stratifying the sample by age, income, and dependency across the tiers of nursing, the study sample was extended to 23–24 interviews per tier, for a total of sample size of 71. Each interview lasted between one- and 2.5-hours. Interviews were conducted between April 22, 2015 and July 24, 2015.

The influence of the PI’s race and country of origin were sources of concern prior to interviews but they did not present systematic challenges, with one possible exception. Indeed, her background, open interview style, and apparent impermanence in the city may have led participants to offer more detailed interpretations of their experiences than they would have otherwise. Other white, English-speaking nursing researchers have found that openness contributed to participants’ comfort [7]. Still, the PI’s race and country of origin may have kept participants from divulging information about intimate partner violence (IPV). Participants volunteered responses in interviews; they were not asked directly about IPV and none raised IPV as an issue impacting them directly. Statistically, however, it is unlikely that all participants have no personal experience of IPV.

The best paid of three tiers of nurses in South Africa, registered nurses (RNs) have completed at least four years of publicly- or privately-funded training in general nursing and, for those with nursing diplomas, midwifery. Enrolled nurses or staff nurses (SNs), the middle tier, have passed two years of nursing education, largely from private training programs for which nurses and their families pay themselves. Enrolled nursing auxiliaries (ENAs) with one year of education, usually privately funded, constitute the third tier.

### Definitions

The study operationalizes conservative definitions of dependents to map networks of dependency. “Internal” dependents live in a nurse’s immediate household and the status is limited to children under the age of 18 and adults who were neither working for pay nor receiving an old age pension or a disability grant (both available through the South African state). The Old Age Pension and the disability grant paid R1,410 ($118) in April 2015, the time of the interviews [35]. All adults in the immediate household who earn income, as reported by the nurses during the interviews, regardless of their earnings, are excluded from the count of internal dependents. The definition of internal dependency is restricted for two reasons. First, if income is not pooled, as is common in developing countries, the primary dependency relationships tend to be between children, the elderly, and the ill or disabled and women. Second, the exclusion of income earners from the dependency figures helps generate conservative estimates by discounting household members who may be self-supporting.

External dependents include those a nurse specified as recipients of monthly remittances or in-kind transfers, such as groceries. If monthly remittances were made for groceries for an entire household in the nurse’s household network, all members of the household are considered external dependents. External dependents may therefore include individuals earning income, as nurses may send money directly to those who are employed or make transfers to households in which they reside.

In the remainder of the paper all rand/dollar conversions are calculated at the exchange rate at the time of the interviews of R12 to US$1. All values are medians unless otherwise specified. Abbreviations are as follows: registered nurse (RN), staff nurse (SN), enrolled nurse auxiliary (ENA).

## Results

### Demographics, background, household structure, and time use

The 71-nurse sample includes about equal numbers of nurses in each of the three tiers: 24 RNs, 24 SNs, and 23 ENAs. While the mean age was 40 years, RNs tended to be older (median: 49 years) than SNs (35 years) and ENAs (33 years). Two-thirds were between the ages of 24 and 44 years (Table 1).

**Table 1:** Age of participants.

Age group	N	%
24–34	29	40.8
35–44	19	26.8
45–54	15	21.1
55+	8	11.3
Total	71	100.0

All participants self-identified as black African women with the exception of one nurse who self-identified as coloured (the accepted South African term for mixed race). All were born in South Africa.

The nurses came from a wide variety of socioeconomic backgrounds and household arrangements. Of those who volunteered information (n = 48), the majority grew up in a household with their grandmother (35%) or their mother only (25%), while 40% grew up with two parents. Children growing up with a grandmother or an aunt remains a common family form in South Africa, whether or not a child’s parents are living or married [36,37]. The practice is known as child-leaving or child-sending.

While almost all SNs and ENAs worked in wards on 12-hour shifts, RNs are divided between those who work in 12-hour shifts in wards and those who work from seven in the morning to four in the afternoon, Monday through Friday, in hospital clinics or administration. The majority of the participants, 79%, worked 12-hour shifts in hospital wards (Table 2).

**Table 2:** Paid worksite and schedule.

	N	%
Hospital ward (12-hour shifts)	56	79
Hospital clinics (seven a.m. to four p.m. from Monday to Thursday and seven a.m. to one p.m. on Fridays)	13	18
Administration (seven a.m. to four p.m. from Monday to Thursday and seven a.m. to one p.m. on Fridays)	2	3
Total	**71**	**100**

Almost all of the nurses belonged to a union. Over half (n = 38; 54%) were in the Democratic Nursing Organization of South Africa (DENOSA) and another 30% (n = 21) were in the National Education, Health and Allied Workers' Union (NEHAWU). Five nurses were in the National Union of Public Service and Allied Workers (NUPSAW) and two were in the Health and Other Services Personnel of South Africa (HOSPERSA).

On a workday, ward-based nurses spent their 12 hours outside of the hospital sleeping (mean: 6.6 hours), doing unpaid work such as cleaning and cooking (mean: 2.8 hours), commuting (mean: 2.0 hours), and in leisure (mean: 33.0 minutes). Nurses without children living with them had about an hour of leisure time on a work day, while those who did had 22 minutes.

Three-quarters (n = 53) of the nurses have children under the age of 18 (Table 3), but not all have their children living with them. Twenty percent of the nurses (n = 14) have a child being raised in a household other than their own, including 10 of the 23 ENAs, three SNs, and one RN.

**Table 3:** Nurses and children.

	N	%
Nurses with children, age<18 (may or may not be living with her)	53	74.6
Nurses with only adult children, age>18 (may or may not be living with her)	13	18.3
Nurses without children	5	7.0
Total	**71**	**100**

Nurses live in a variety of household formations: with partners and children, with children without partners, with partners and no children, with relatives, and alone (Table 4). About half of RNs and SNs live with a partner, while a plurality of ENAs (35%) live without a partner but with a child.

**Table 4:** Structure of nurses’ immediate households by tier of nurse.

	N	%
**Living with a partner and at least one child under the age of 18**	**27**	**38.0**
RN	11	40.7
SN	11	40.7
ENA	5	18.5
**Living without partner and with at least one child (age<18)**	**16**	**22.5**
RN	4	25.0
SN	4	25.0
ENA	8	50.0
**Living alone**	**12**	**16.9**
RN	3	25.0
SN	4	33.3
ENA	5	41.7
**Living with related adults (all over 18) and no children**	**11**	**15.5**
RN	5	45.5
SN	3	27.3
ENA	3	27.3
**Living with partner, no children under the age of 18**	**5**	**7.0**
RN	1	9.1
SN	2	18.2
ENA	2	18.2
**Total**	**71**	**100.0**

Nurses’ one-way commute times range from 10 minutes to 112 minutes (mean: 58 min.; median: 60 min.), most via public transportation (63% in minibus taxis, 10% by bus). The commute is both a source of stress and a period during which nurses experience distress. Commutes in minibus taxis were stressful due to expense, safety concerns, and unpredictable timing, which can present childcare challenges in addition to rendering timely arrival to work and home difficult. Using a car to get to and from the hospital, a mode of transport used by half of RNs (n = 12) and about 20% of SNs (n = 5) and ENAs (n = 4), is seen as an enormous relief by those using public transport.

For nurses who do not live in their household alone, the time during their commute is typically the only period of a workday in which they are not obligated to serve the people around them, be it in the workplace or the household, meaning they have time to themselves. However, 68% (n = 48) reported thinking about ways in which they were worried about others or were dissatisfied with their lives related to debt, family dependency, exhaustion, and responsibilities, thus the time was experienced as a period of distress. Others explained that they play games or listen to music on their phones to try and avoid thinking about problems, in one case in order to keep from crying in public.

To summarize, the participants are black women nurses, most between the ages of 25 and 55. Over 90% have children of any age and 75% have children under 18. Their households vary in form: 38% live with a partner and at least one child under 18 and about one-quarter live with children under 18 without a partner. The latter figure underestimates “single mothers” (a concept with questionable utility in this context) because another 20% have children that are living away from home. Some of those nurses live alone while others live with only adults. Most participants work 12-hour shifts in hospital wards and many spend two hours commuting on public transport on workdays. Nearly all are unionized.

### Finances: Income, expenditure, and debt

#### Income

All 24 RNs grossed at least R12,000 ($1,000) per month. ENAs grossed between R8,000 ($667) and R10,000 ($833), and SNs grossed up to R14,000 ($1,167). The median gross for RNs is R20,200 ($1,683). However, expenditures come out of nurses’ take-home pay, for which medians are R7,700 ($642), R9,000 ($750), and R12,000 ($1,000) respectively (Table 5). Deductions were taken for taxes, pension, and optionally for medical aid, retirement saving, and insurance policies. Overall median take-home pay is R9,000 ($750) per month.

**Table 5:** Monthly income in rand, rounded to the nearest R500 (U.S. dollars at R12/$1)^a^.

	Gross Income		Take-home pay	
	Mean R US$	Median R US$	Mean R US$	Median R US$
RN Specialty^b^ (n = 5)	R33,600 $2,800	R33,000 $2,700	R22,000 $1,800	R22,000 $1,800
RN General (n = 19)	R19,300 $1,600	R18,500 $1,500	R13,000 $1,000	R12,000 $1,000
SN (n = 24)	R11,500 $950	R11,400 $950	R9,000 $750	R9,000 $750
ENA (n = 23)	R9,000 $750	R9,000 $750	R7,500 $625	R7,700 $642
Total	R13,700 $1,140	R11,000 $900	R10,000 $800	R9,000 $750

^a^ Gross pay, net pay, and deductions were self-reported in interviews; several participants had paystubs with them. The figures were evaluated for internal consistency against documentation detailing the pay levels for nurses, and against monthly spending diaries completed by a subset of nurses [38].

^b^ RNs are divided into generalists and specialists because the pay varies considerably between the groups [38]. However, some nurses with specialty training are in generalist positions, and some with specialties were among the most indebted, therefore the RNs are grouped together elsewhere in the paper.

Income was not typically pooled at the household level; only one nurse had a joint bank account with her husband. Instead, each earner tended to cover a set of expenses determined, in part, by the gendered norms and power relations described below.

#### Expenditure

Over 90% of the nurses held debt from bank loans (excluding mortgages), credit cards, and/or department store accounts. For those who made them (n = 65), debt payments on loans, credit cards, and accounts were a major monthly expense (median: R2,000; US$167). On average debt payments took up 18% of nurses’ gross pay and 24% of their take-home pay. For 43% of those with debt, these payments were their largest expenditure per month. Debt is explored in more detail below.

Housing was an expense for half of the nurses (n = 35) while the other half lived in family homes, in their own homes for which the bond had been paid off, or lived with a partner or family member who paid the rent or bond. Those who paid for housing either rented (n = 22; median: R1,700/month; $142) or paid a bond (n = 13; median: R3,800/month; $317) (Table 6). Note, however, that all nurses received a housing allowance (included here in gross pay and take-home pay because it is added onto their paychecks) of R900 per month, the cost of nurses' housing in government-owned properties. Those who paid rent (n = 22) sent more money in remittances and made larger debt payments than those who paid for a bond (n = 14). For ENAs (n = 7) and SNs (n = 9) who paid rent, their largest expenditure each month was on debt payments.

**Table 6:** Median monthly housing expenditure, in rand (U.S. dollars at R12/$1).

	Rent		Bond	
	n	R US$	n	R US$
**RN**	6	R3,200 $267	7	R4,200 $345
**SN**	9	R1,500 $125	6	R3,700 $308
**ENA**^a^	7	R900 $75	1	.
**Total**	22	R1,700 $142	13	R3,800 $317

^a^ There is one ENA who pays a bond. The datapoint is excluded for the sake of maintaining the participant’s privacy.

Table 7 summarizes median expenditure on major monthly expenses by tier of nurse. Note that not all nurses have each expense (e.g. not all send remittances, not all pay rent etc.), therefore the data cannot be aggregated into an estimate of overall monthly spending.

**Table 7:** Median monthly expenditures by tier of nurse.

	Remittances		Groceries (incl. toiletries)		Transportation		Utilities (electricity, water, phone)		Policies (insurance)	
	n	R US$	n	R US$	n	R US$	n	R US$	n	R US$
**RN**	12	R1,350 $110	24	R2,400 $200	24	R1,000 $83	16	R704 $59	16	R620 $52
**SN**	17	R1,300 $108	24	R1,000 $83	24	R636 $53	21	R400 $33	10	R221 $18
**ENA**	20	R1,465 $122	23	R1,250 $104	23	R610 $51	21	R254 $21	10	R220 $18
**Total**	49	R1,300 $108	71	R1,325 $110	71	R620 $52	58	R475 $40	36	R400 $33

Remittances to relatives (n = 49; median: R1,300; $108), groceries (n = 71; median: R1,325; $110), transport (n = 71; median: R620; $52), utilities (n = 58; median: R475; $40), and insurance policies (n = 36; median: R400; $33) are nurses’ other common expenses.

Additional monthly expenses related to children included local childcare (n = 14; median: R1,000; $83) and school fees (n = 35; median: R1,250; $104). Other spending, some of which was substantial, included remittances that are not monthly, payments for healthcare, legal fees, cable television, and tithes for church.

Nurses, especially those living in single-income households, are concerned about home ownership, which is widely seen as a necessity for raising children but as unaffordable for SNs, ENAs, and some RNs. Nurses repeatedly noted their ineligibility for Reconstruction and Development Program housing. They estimated that a salary or combined household income needed to be at least R14,000 per month in order to secure financing for a bond from a bank.

Nurses were also concerned about their ability to retire. Even those who had paid off their bond and built second homes in rural areas for retirement expressed worry about their adult children’s ability to pay levies and utility bills in homes or apartments owned by the nurses. A 63-year old ENA (R1I28) describes her concerns:

*So*, *when I'm 65 [I want to retire]*. *And then*, *I'm stressed that*, *"What is going to happen to this flat*?*”…[I]f I was doing well*, *if my kids were working*, *yes*, *I would retire*. *But now*, *because they are not working*, *I still have to work and pay the levy and pay for food for them*. *I am stressed; worried about it…*.*I’m going to move back [to another province]*. *But*, *you know*, *my son*, *if he is still looking for a job and they still need to stay in the flat…That also stresses me*, *because if they're not working*, *how are they going to…I’m still going to have to pay the levy even while I'm in [my other home]*…

Financial concerns and familial concerns tended to be interrelated, as in the cases of home ownership and raising children, one’s ability to retire, and debt management.

Most nurses’ spending on common expenses (housing, remittances, transportation, groceries, utilities, insurance, school fee, childcare etc.) exceeds their net income prior to accounting for payments on debt (or savings). The average ENA spends 1.5 times her net pay, the average SN spends 1.3 times, and the average RN spends 1.1 times, suggesting that nurses are accruing debt.

### Debt

*These days nurses are in debt*, *we have family problems*, *we are divorcing because of the long hours that we are working*. *You see*! *We are working night shifts and then our husbands are disposed to cheat*. *You see*, *then we end up being*, *like*, *vulnerable [to HIV infection]*. *You see*? *And then the pressure again at work*, *you must work hard*. *So*, *you burn out*. *And you will find nurses that are working a double shift*! *I'm sure you are aware of it*! *For example*, *they go somewhere else trying to get money*. *So*, *what do you expect*? *If that is what a nurse is forced to do then what do you expect from that nurse*? *You have this burnout syndrome*. *Like*, *you get angry*. *You are exhausted*. *You don’t have time for yourself*. *These other nurses at work*, *you work here 12 hours*. *You work at another hospital*. *Tomorrow you must be at home—maybe*. *You see*? *It’s a problem and I don’t know how it can be solved*. *Some of them*, *it’s us [the nurses who are working with those who are doing double shifts] who put ourselves in unaware*. *You see*. *This debt thing*, *it’s serious*.

—SN (R2I36) in her early 40s who lived with her husband and four children. She spent 24% of her monthly take-home pay on debt from a loan, her accounts, and a credit card.

Debt was a source of considerable stress. All but two nurses carried some debt and the debt burden from loans and on department store accounts was substantial. Monthly debt payments constitute up to 40% of a nurse’s gross pay per month, and up to 67% of take-home pay, with the average being 19% of gross and 24% of take-home (Table 8).

**Table 8:** Median monthly expenditure on debt by of nurse.

	n	R US$	Median debt payment as a % of gross monthly income	Median debt payment as a % of monthly take-home pay (excl. taxes, pension deduction, medical aid, union dues)
**RN**	20	2600 $217	13	20
**SN**	24	1600 $133	13	19
**ENA**	23	2050 $171	24	29
**Total**	67	2063 $172	19	24

At least two-thirds (n = 48) of the nurses held debt in department store accounts (median monthly payment: R685; $57). Among those, the median estimated total owed on accounts per nurse was R5,400 ($450). Loan indebtedness (median monthly payment: R1,835; $153) was common (n = 49) and often due to family breadwinner responsibilities including renovating or building parental homes (n = 13), paying for siblings’ and children’s educations (n = 9), paying for a car (n = 6), renovations to own home (n = 5), own education (n = 4), paying off other debt (n = 4), and paying for funerals (n = 2).

Debt on department store or grocery store accounts was a source of stress and guilt because it tended to be interpreted as indulgent spending on one’s self, while indebtedness from loans tended to be understood as pertaining to familial pressures or gifts to the family. In practice, the relationship between debt from loans, credit, and remittances was more complex. Account debt was directly related to loan debt and remittances, therefore to familial pressures: nurses sometimes feel forced to incur debt on accounts, which included some grocery store accounts, to make up for income they must spend on loan payments or remittances. The same SN above (R2I36) reported a great deal of financial pressure from external dependents. She explained:

*[S]ometimes I sacrifice*! *I’ll give them that money though I don’t have it*. *And I’ll go and get a credit somewhere*. *It affects me…I can’t say I’m coping*. *I get*, *like*, *emotional*. *Like*, *I ask myself why things are like this*.

Debt caused some nurses to experience anxiety around the 15th of each month, their payday. A married ENA (R1I30) in her late 20s described her feelings:

*From the 1st to the 13th [of the month]*, *I’m ok*. *But on the 14th*, *oh*, *that’s when the trouble starts*. *Payday makes me anxious because of my debts*. *Pay comes in and you are happy*, *“Okay*, *this is my salary*.*” Then it goes down [once it is deposited due to automatic deductions for payments] and you wonder*, *“What’s happening*?*” and when you add two plus two you are left with nothing*. *So*, *I become anxious*. *…[W]hen you add two plus two*, *[I] still have to send money home for my other child*. *I still have to help my husband with some of the things in the house*. *I still have to keep money for transport*.

*[Payday] is a reminder*, *“You know*, *I’m getting paid*, *but I can’t even buy myself a little thing*, *all my money will just go to pay my debts and stuff*.*” That’s the problem*.*…I’m upset about not being able to provide for my kids the way I wanted to*. *Like*, *you know*, *growing up and saying*, *“Oh*, *my kids will have x*, *y*, *z*.*” But now that I’m older*, *I have kids*, *I’m working*, *I can’t do what I wanted to do for them when I was growing up…I didn’t want my kids to grow up like I did*. *I wanted to put them in good schools*. *So*, *that’s something that is painful to me*.

Another ENA (R1I27) fantasized about the day she will be able to pay off her debts, which included a loan she took to build a home in another province for herself and her sister, who is raising the nurse’s children. She paid R2,140 ($178) per month for the loan, about one-third of her take-home pay.

*I dream of that*. *One day I will have money*. *I wish I had money and could pay all of them*. *And be debt-free*. *Once I have money…I’m making sure that I go to all the shops and pay them*. *“How much do I owe you*?*” “They’ll say*, *“No*, *the due amount is so much*.*” But I can say*, *“Ok*, *that’s fine*, *you can take it*. *You can take it*, *you can take it*.”

*Sometimes I am debt-free*! *Some years I am debt-free*. *It depends on the year*, *on inflation and everything; if we are in a recession*. *It's very tough this year because my brother is in school*, *my kids are in creche*, *my fiancé is retrenched*. *It’s an overburden*.

In addition to the loan she is paying off, she owed an estimated R10,100 ($842) on various department store accounts and was stressed by the account representatives who called her on the phone. She avoided answering calls from numbers she did not recognize but then worried that she may miss a call from her children or from a hospital closer to home where she applied for a job.

ENAs spent a median of R2,050 ($171; mean: R2,000; $167) on debt per month, about 29% of their take-home pay. RNs spent a mean of R3,200 ($267) and median of R2,600 ($217), or about 24% or 20% of their take-home pay.

### Dependency

Dependency is calculated by adding the number of dependents a nurse supported and putting that number over one. The one represents the nurse and her salary. If the top number were three, it would mean the nurse supported three people as well as herself, for a total of four. For all earners in South Africa, the average dependency ratio is 1.55 [3]. Using conservative assumptions and strict definitions, the dependency ratio for the nurses is far higher at 5.24.

It is likely that all of the people living in nurses’ immediate households benefitted from nurses’ incomes, but for the purposes of this study adults living with the nurse are only counted as dependents if they did not earn income or receive a monthly government grant of over R1,000. Almost three-quarters of the nurses (n = 52; 73%) supported dependents internal to their households, including 84 children and 46 adults in addition to the nurses themselves. Nurses who support dependents inside their households have an internal dependency ratio of 2.5.

The majority of nurses also supported external dependents. People outside of the immediate household might depend on income earners to send money, buy groceries, pay for household construction or repairs, pay for medical insurance, pay for funeral policies, contribute to funerals and weddings, or pay for costs of education. Counting only *monthly* remittances from nurses to individuals in households other than their own, 49 of the 71 (69%) nurses sent money to at least one person at least once per month. Those 49 nurses supported a total of 92 children and 150 adults externally each month, an external dependency ratio of 4.94. Fourteen nurses were supporting their own children (23 of the 92 external children) in another household. The other external children tended to be nurses’ sisters’ children. Many adult external dependents were unemployed working-age relatives.

The case of an SN (R1I11), a divorcee with no children who lived alone in an apartment in Johannesburg, demonstrates how external dependency can take shape and how it can affect a nurse’s own well-being. She supported six external dependents, one child and five adults, none of whom were working for pay. Her father had been injured at work and the family was without savings, so she had become the breadwinner several months prior to the interview. She explained the situation through tears:

*I think about my family all the time*. *I think about what I need to do for them*. *Most of the time [my father] is asking for money*. *He will act like I don't want to help them*. *I cut everything off on my side*. *I survived on bread and tea for these last two months*. *I’m not taking it well*. *I talked to a manager to see if she can assist with accommodation in nurses’ housing because I can’t afford to send [money] back and pay my rent*.

Only two of the 71 nurses had no internal or external dependents.

### Gender norms and gender roles

*I’ve been a slave for a long time to my husband*.

—44-year old ENA (R2I22), as she explained why she was seeking a payout from her husband’s pension in their divorce proceedings.

Gender norms were explored in detail. Norms are enforced in marriage, but in practice the norms impact all women, especially through responsibility for childcare.

Gender norms and roles are themselves a source of resentment, as noted by an RN (R1I25), a 49-year old married mother of five, and parent to a relative whose mother passed away:

*I dislike the fact that I’m supposed to be the one who does most of the work*. *But then it will be said that it’s because…*.*in a society*, *I don’t know*, *most of the work is done by us [women]*. *I dislike the fact that most of the things that are supposed to be done are supposed to be done by women*. *Because society dictates that that’s what you need to do*: *get married and have children and look after your husband*. *Do this and do these chores*. *I don’t like that fact that I should be vetted*. *Because as it is*, *I do the housework and I come here and put the bacon on the table*. *And I dislike that fact*.

Of particular irritation was that the RN’s husband had fallen out with business partners, which caused the family to lose their home and two cars, and left her to maintain the household. She felt trapped in nursing by her husband’s unemployment, which she interpreted as a function of his unwillingness to work under a boss. She felt that she was taking on both the devalorized role of the unpaid worker and the overvalorized role of the breadwinner. For nurses in our study, role conflict is a source of stress, as is the associated workload.

The work assigned to women by traditional gender roles and gendered expectations was also a significant source of stress. A married 35-year old SN (R1I21) and mother to three children in her household, all under the age of five, who works 12-hour shifts explained:

*All I can say is that he is relaxed when I come back from work*. *I must dish up [serve him dinner] and then take his plates [clear the table]*. *And then he’ll tell me*, *“get me water*.*” And eh*, *I’m also coming from work*. *And I must go and fetch that water*. *It’s like that*.

The work, and the fact that it is often not shared, can be especially stressful for nurses who are pregnant, such as a 31-year old ENA (R1I27), who pointed out that gender norms may be stricter for black women than for white women in South Africa. She described her experience:

*As a woman*, *you have to take care of your family…If you're a man you also have to*, *but not like if you're a woman*. *It’s not MORE*, *like it is for women*. *Women are working more than men*. *You can find that men and women are working together [in paid work] but a man will just come home from work*. *But the woman will come from work and must start doing house chores and cook*. *The man*, *you will find him sitting like this*, *reading the newspaper*. *That’s really stressing me*!

*You know*, *the black people*, *they are not the same with the white people*. *They are…they are…what can I say*? *He will say to you*, *"I can't do that*, *this is a woman's job*, *that thing*.*" Even to cook for himself*, *he can't do that*, *he waits for when you knock off [finish your shift] and do everything–to wash the dishes*, *everything*. *I mean*, *from the child’s homework*, *to ironing his shirt*! *He can't even iron his shirt*!*…I think it's the way that we grew up*, *and saw it at home*, *"My mother does everything*, *so when I'm male*, *they can't do anything*. *It's my wife who can do everything*."

*It's like*, *the last day when I was pregnant*, *I was staying with my boyfriend*. *You know*, *I would knock off here at the hospital*, *I was driving myself*. *He was staying at [township about 40 kilometers away]*. *Knock off here*, *and go [there]*. *He can't even wash any of the dishes*, *not even just sweeping*, *tidy up at home*, *he can't even do that*. *Just sit and wait for me to do everything*. *And I was like*, *"Yoh*, *this thing is hard for me*, *because you know*, *I'm pregnant*.”

At the time of the interview, the nurse’s baby was six months old; her sister had died in childbirth the previous year.

Nurses suffered from fatigue from the amount of work that they do. One nurse (R1I22) noted, “*Sometimes you just cry because you are so tired*.” For a fortunate few, husbands and relatives violate gender norms and prepare food before the nurse returns home (average time 8:03pm for those working in wards) on workdays. Others, such as this SN (R1I18) were not so lucky:

*If you come from work*, *you have a headache*, *there’s nothing to eat at home…you’re supposed to start to prepare supper… and your kids are looking at you*. *It’s a big challenge to have someone else there and have them not helping*. *And they think you are lying*, *like you don’t have a headache*. *That you don’t want to cook*.

*Q*: *The kids or the husband*?

*A: The kids feel shame for you. The husband is the one that thinks you don’t want to cook; you’re just creating a disturbance*.

*Q*: *How does that make you feel*?

*A*: *Yoh*! *Yoh*. *Bad*.

*Q*: *Bad how*?

*A*: *Because you can’t show the person that the headache really is there*. *And somebody doesn’t believe you*. *It’s so stressful*.

None of the nurses raised domestic violence as a challenge that they themselves confronted, although it is common in South Africa [39]. One nurse’s sister was killed by her husband and another nurse’s father killed his wife. A third nurse moved between households repeatedly as a child due to violence in her parents’ relationship. When domestic violence was otherwise raised in the course of the interviews, nurses tended to describe it as a response to women breaking gendered norms about unpaid work, which they linked to the practice of men paying lobola or bride price. Lobola has complex meanings for women and men in South Africa [12]. Nonetheless, a 30-year old married RN (R2I35) describes the relationship between lobola, work, and the construction of norms:

*A*: *[T]hey know that men cannot wash the dishes or cook*. *A wife must do that*. *It’s like that*. *And men cannot do the laundry*. *My wife must do that*. *They know that—strictly that*. *They do nothing; they just come and provide everything [financially]*. *If they can change that mentality to say*, *“No*, *I can do the laundry also*, *I can cook for my wife*, *I can help her to clean*, *I can help with the dishes also*,*” that will be better*. *At least have a hand to say*, *“I can iron*, *I can do ironing for my wife*.*” It makes them feel like they are not man enough when they do those things [cooking*, *cleaning*, *or ironing]*. *Some men will tell the man*, *“my wife does this*, *you’re doing this but you’re not supposed to do that*.*” Peer pressure also outside [keeps men from doing unpaid work at home]*. *They say you have to stand up to your wife*.

*Q*: *So*, *they think that they can’t do this work*, *but they can*?

*A*: *But they don’t want to because when you get a wife*, *your wife must do everything for you*. *Because you paid lobola for this*. *In our tradition you have to pay lobola*. *You have to take out money or cows to go to that family to ask that woman to be your wife*. *And when you have done that and the woman goes to stay with him*, *they say now this man has taken cows for you*. *You know the duties of a wife*: *you wash for your husband*, *you clean*, *you cook*. *You even wash his feet*. *You wash his feet*, *wash his face*. *When you go in that new family of yours*. *The elders will tell you “do this*, *one-two-three*, *one-two-three*, *one-two-three*.*” You don’t want that man to come back and say*, *“I want my cows back because this one didn’t do the job that she is supposed to do*.”

A married SN (R2I29) in her mid-40s described a seemingly stereotypical marital relationship, which may include violent reinforcement of gender roles and the work they entail:

*A: But he [the husband] will tell you, if you say*, *“Get up and do things yourself,” “I’m the man here*. *I married you so that you would do things for me*. *I paid lobola*. *I bought you so that you must do things for me*.*” Some of the men say such things*.

*Q*: *If she said no…*?

*A*: *Some of the men*, *they are going to beat her*. *They are going to beat her for that…and say*, *“You better take your things and go back home to your family*.”

Our interviews supported the notion that, beyond housework, gender norms that dictate that care work is “women’s work” increase dependency on women. The norm is especially stringent with respect to children: women have primary responsibility for supporting children, usually both financially and for their care. An RN (R1I25) explained:

*[W]ith raising my kids*, *I’ve got five very good children*. *Well-behaved*, *not spoiled brats*. *And I did that on my own*! *Because my husband is kind of*, *um*, *he’s a traditional kind of a black man…like*, *he’s not involved in the raising of the kids*, *like I would want him to do*.

Primary responsibility for children holds for women regardless of whether they are partnered. Most household expenses and those related to childcare were shouldered by nurses, although their primary responsibility did not preclude contributions from children’s fathers. Some partners were supportive financially. Other nurses described passive or active resistance when they asked partners to bear some of the expenses of caring for children.

“Child-leaving” is a common strategy among nurses, due in part to gender roles, nurses’ shift schedules, and the availability and cost of childcare, particularly during unsociable hours. While leaving a child to be raised in a household that belongs to a nurse’s mother, mother-in-law, or sister can be a relief from the stress and expense associated with childcare, it can introduce another set of concerns. Child-leaving is a source of emotional and psychological distress for some nurses who would prefer to live in the same household as their children. One nurse (R1I27) described it as an “emotional struggle”:

*It’s difficult*. *My kids are growing up without me*. *Sometimes they are sick or they just want to be next to their mother and their father*. *And it’s hard for them to reach us*. *It’s very*, *very difficult*. *Even when I’m at home like on Sunday I have to get them dressed to go to Sunday school and then I leave before they come back*. *Because if I leave when they are in the house they will cry*. *It’s very difficult for me*.

It is disproportionately ENAs who have children being raised in other households. The strategy is used by 10 ENAs, three SNs, and one RN.

Upon reflection, some nurses—even RNs whose household incomes afforded a nanny—said they would have chosen a different profession if they had known how incompatible nurses’ schedules would be with motherhood. In response to the question of whether she found having children rewarding, one RN (R1I25) with two adult children, two teenagers, and another child in elementary school responded:

*It was*, *but knowing what I know now*, *would I do it [again]*? *Like knowing what I know now*? *I would do it differently*. *Not in this job*. *I would have kids but not in this job*. *Because this job*, *it has been torturous you know raising kids doing 77s [12-hour shifts] and doing night duties*. *It was very*, *very difficult for me*. *But it was rewarding being a mom*. *But the work was too much*.

Another RN (R1I33), a single mother in her late 30s, describes how the shift schedule and fatigue make it a challenge to give her young teenaged son the attention that she believed he needs:

*The hours are killing us…We are able to provide bread and butter for our families but our children are suffering because we are never at home*. *Yes*, *we can provide money*. *But I’m not at home*. *And our kids want us at home*, *they want to feel our presence*. *My son wants to tell me everything about school and soccer practice*. *But sometimes I’m so tired that I’m not even listening to what he’s saying*. *He’s telling me all these stories and I’m so tired I don’t even hear what he’s saying to me*. *At the end of the day*, *if you don’t look after them someone else will look after your child for you [referring to gangs]*. *Many nurses’ children do drugs and they can afford them because of the money the nurses earn and give to their kids*. *We think that giving them money will solve problems but it doesn’t*.

#### Relationships and money

Nurses reported that gender norms contribute to complex relationships loaded with personal and financial ramifications, which can be a major source of distress. Women workers are expected to provide financial support, but their contributions and associated difficulties may go unacknowledged, which can cause them to feel unappreciated. The 32-year old ENA (R1I11) with only external dependents discussed above explained through tears:

*I feel like no one is appreciating…At times I feel like [my contribution] is not recognized because I never had anyone coming to me and saying thank you*. *Appreciating me being around [when I go home to my parents’ house]*. *Or asking me if I am managing*, *or if I am still doing fine*. *And I even asked about it and the answer I got from my father was that he said when he looks at me*, *he sees me as somebody who doesn’t need to be looked after*. *I can take care of myself*. *That is the answer he gave*.

Debt, dependency, work, and gender norms are interrelated. Another ENA (R1I35) in her early 30s supported an unemployed adult brother and an unemployed fiancé in her home (originally her mother’s home), a high school-aged brother who lived elsewhere, and her own two-year old, who lived with her fiancé’s mother. She described her distress while explaining how she ended up with debt, originally from a loan that she took out to pay for her education but which ended up being spent on other household demands.

*No*, *it's not stress by [paid] work*. *It's [paid] work [too]*, *but the more [pressing] stress that I have is now I’m alone [since my mom passed away]*, *no one’s helping me*. *It's the thing that is stressing me*. *Now I'm alone [in supporting the household]*, *I have to take care of my* … *son*, *my little brother*, *my*, *the other brother*, *and my boyfriend again*. *It’s financial…everything’s on my shoulders* …*I was the breadwinner even then [while my mom was alive]*. *…[A]nd now*, *the whole [family] is looking at me…I think maybe if I can get this loan paid off*, *maybe I'll stress less*. *Because the loans are pulling*, *and I have to go and pay for other stuff*, *I have to help my family*, *my [mother] in-law is sick*. *I have to do this and that*, *do this and that… They push from that side*, *that side*, *that side*. *And then you end up using money for the wrong reasons*. *It's taking a long time to pay it off*. *It's like losing weight*. *It's not easy*.

Several nurses (R1I1, R1I5, R2I26, R2I17, R2I31) are in debt because they took out loans for family members (three husbands, one mother, and one brother) for the purpose of starting or expanding a business. Another (R2I22) took out a loan to buy car parts for her husband whom she expected to pay her back but who never did. At least two (R2I8 and R1I1) took out loans to pay off husbands’ or other relatives’ debts. Given women’s primary responsibility for supporting children financially, the loan payments that are deducted from the nurses’ bank accounts—in this case for loans that were for other people—can cause instability and be a source of discontent. An RN who made a monthly debt payment for a loan she took out for her husband, a payment that took up 67% of her take-home pay explained:

*There are things I have to do…currently*, *as I said*, *he’s starting his own business so I can’t even say I can start my own business*, *you know*. *I have to make sure that there’s food*, *the school fees are paid*, *you know*, *things like that*.

A common division of financial responsibilities was that women living with a partner paid for day-to-day household spending and their partner paid the monthly rent or bond on the house. This division of fixed versus variable expenses suggests that men sometimes had money left over that they were able to spend on themselves, other partners, or save, while the nurses found that their expenses tended to expand, such that many were unable to save. Further, paying for the house put some men in a position of power that they wielded against their partners. A 29-year old SN (R2I13) who lived with her husband and her two-year old child explained that she felt that her contribution to the household went unrecognized, and that she would like to leave her husband but felt trapped in her relationship:

*OK*, *the thing is that in the house that we're living in*, *it's a joint bond house*. *He's the one who's paying*. *The money's deducted from his account*. *So*, *he always*, *he reminds you that*, *"this is my house*,*" you know*? *I just think how can I get out of this situation or move out from this situation*, *you know*? *Or buy my own house*. *I'm getting tired of this*. *And I do a lot*, *you know*? *I spend my money …it's just not appreciated*. *So*, *that's something that's stressing me*. *<begins crying> I can’t escape this*. *I can’t escape it*.

The nurse continued, however, to explain that her salary would not allow her to buy her own house. She believed she would be ineligible for a bond on her own salary.

A highly-paid RN (R1I17) in her late 50s who supports two unemployed adult children and a grandchild was left with an enormous amount of debt when her husband walked out on the family years ago. She said:

*He always says it’s my fault*. *Everything has been my fault*. *It’s because*, *now*, *because he was not working*, *I wasn’t giving him money*. *I was always caring about the kids*, *doing everything for the kids*. *What was I supposed to do*? *He says that’s why he just left because now I couldn’t afford*, *now I stopped giving him money*. *Then he always says it’s my fault*, *that’s why things went wrong…*.*he also left me with debts*. *… Sometimes he said he’s going [away] on business but he doesn’t have money*. *I have to borrow money*. *…Sometimes I borrowed for him and he’d end up not giving the money back and I ended up in a lot of debt*.

Nursing may be experienced by some at some times as an occupation forced on them by dependency or debt, while it has enabled others to leave relationships. An SN (R2I16), a mid-30s mother of two who had previously been entirely financially dependent on her husband described her feelings after her divorce was finalized in the month prior to the interview:

*I am happy*. *Now I am happy <laughing> and it took me a long time*! *It took me a long time to get out of this marriage*. *I wasn’t happy at all*. *But it took me a long time to get out of this marriage*. *Why did it take so long*? *I don’t know*. *Maybe it’s because of my background*? *My mother is not financially stable*. *I don't have a father*. *I don't know where to go with my kids*. *I don’t know who’s going to take care of me and my kids*. *So*, *I stayed there*.

A 34-year old RN (R2 I32) mother of one also found that nursing offered some economic autonomy:

*Women work harder than men both for money and at home doing chores*. *Men don't take responsibility*. *You can't leave your child with the father*. *You can’t trust that your child is safe with him*. *I contributed more than the father did*. *The relationship was oppressive too*. *I couldn’t go out with friends and I couldn’t leave my daughter with him*. *There is disproportionate responsibility*. *Nursing was a way out of the relationship*.

Still others find it difficult to leave relationships because of marriage laws that are understood to divide assets and debts equally among former partners upon the dissolution of a marriage. An RN (R1I8) in her 50s with two children explains:

*In marriage*, *once you get married in community of property*, *it’s a problem*. *Because most of the times the African men are earning*, *some are earning*, *but when coming to contribution in the family*, *it’s not much*. *The women are the ones that are…pay for the kids to go to school*, *buy groceries*, *save some money…so this marriage in community of property is another problem or challenge*.

*Q*: *Community of Property*?

*A*: *Every woman now has regrets about getting married in the community of property*. *With my husband*, *he’s not working*. *And once he’s divorced*, *he gets half of what [I have]*.

*Q*: *Do you think a lot of women feel this way*?

*A*: *They normally discuss…it’s a problem everywhere*. *They feel the same*. *Much as I feel the same way…According to South African law*, *there’s nothing we can do*. *There are other ways but it’s not easy*, *the process*.*…[I]f you get divorced and he’s got his own debts*, *they will fall under you as well*.

Gender norms that preclude equitable communication about finances between husbands and wives can disempower nurses and add to their sense of being unable to divorce. One RN (R1I25) simultaneously highlighted how legal institutions reinforce this perception, but demonstrated that she also internalized their message about “personal responsibility”:

*My pastor is [foreign] and we talk about the finances*. *And like you asking me*, *like*, *“How much does your husband earn*?*”…She can’t imagine being married to a man and not knowing his financial status*. *That’s what she tells me*, *like*, *“Brenda*,^*a*^ *how do you live in a marriage like this*? *If I didn’t know how much Paul made…” I’m like*, *“It’s the way it is Jan*. *It’s Africa*!*” and she is laughing at me <laughs>*.

*Q*: *Does this leave you unprotected if you try to divorce*?

*A*: *Yeah*, *because like if I’m standing there in the court of law and I say “I don’t know [about the finances]” they are like*, *“You* *ought* *to know*. *He is your husband*.*” … I think it’s wrong*. *It’s irresponsible*. *It’s like you are gambling with your life*. *Like I told you*, *when his company went bust*, *I almost died*. *I saw these people coming and saying sale of execution*. *I had no idea how deep we were in it*. (emphasis in original)

^a^All names changed to maintain participants’ anonymity in reporting.

While the nurse above reported talking with her pastor, and others said they turn to their mothers, sisters, and husbands or partners for emotional support, virtually none of the nurses spoke with their colleagues about their stress. They tended to view distress as a result of challenges in their personal lives, not their professional lives. The professional status of the occupation may itself discourage nurses from revealing what may be perceived as personal problems or weaknesses to each other.

### Physical and psychological manifestations of stress

Nurses’ lives are characterized by intense and complex interrelated stressors. They reported that the pressure keeps them from relaxing and can create a feeling of being trapped in their jobs, in unhappy relationships, and/or in debt. Racing minds, being overwhelmed, and a feeling of never getting on top of one’s work were extremely common.

Some nurses elaborated physical reactions. Over 15% reported experiencing headaches at least once per month. Backaches and muscle tension impact over 20%, including an ENA (R1I35) in her early 30s who supports an adult brother, her boyfriend, and her younger brother and her son in other households. She described how she felt after her mother, a second income earner in the household, passed away:

*I was saying I'm going to be strong, strong*. *Now I have tension*. *I was off [work] again, off sick*. *My muscles…by the upper ribs, they become tense*. *Because even the doctor said*, *"I can feel this muscle. It's pulling now*.*" Even here [in the hospital] it was pulling*. *And then she gave me pills for tension, to take down the tension*. *And then told me not to stress out*.

Nurses experienced a variety of emotional responses to distress: anxiety, frustration, unhappiness, and anger. An SN (R2I36) in her early 40s whose monthly earnings are the primary support for three children and one adult, and contribute to support for six other adults and children outside of her household responds to the question, “When do you experience stress?”:

*Eish*. *Sometimes when I’m at work and the delegation of things…you find that they gave you the very sick babies*. *And then it's a lot of babies*. *And sometimes you work overtime and when you are supposed to be paid there is no money [overtime pay is delayed] even after you have sent the claim form*. *And you were expecting that extra money and it doesn’t come*.

*Q*: *You said you get headaches when you get paid* …*can you tell me how stress about finances affects you*?

*A*: *My body gets tired and then sometimes I get angry easily*. *I’m not happy*. *And you know when you are not happy you become sensitive with everything*. *Sometimes you even cheek the kids [blow up at her children*]

It was striking how many of the nurses responded to the question “How are you?” at the start of the interview with an answer related to their family: “It’s my son’s birthday.…” or “My daughter is graduating soon…” Repeating the question with more emphasis on the word “you” provoked responses ranging from bemused smiles to bursting into tears. While life history interviews may reasonably be expected to be emotionally-loaded, it was surprising that in over 20% of the interviews the participants shed tears. In some instances, tears came when a nurse was describing a specific life event, such as explaining that a relative had recently passed away. In others, they came when a nurse described the pressure that she felt was on her, how she wished she could do more for her family, or how she felt that what she was able to do was not appreciated.

Chronic stress may compromise nurses’ mental health. One RN (R1I25) said she felt the "most sane” when her mind stopped racing, “*…when I’m not thinking about the worst-case scenario and what there is for me to get through*.” The interview continued:

*Q*: *When you say that you’re surprised you haven’t gone insane*, *what does “insane” mean*?

*A*: *Like when you’re so mad*, *when you’re so very*, *very*, *very*, *very*, *very angry*. *<grabs table>*. *Like if there was a mountain you would stand on the top and go*, *like*, *“aaaaa*!*” <little frustrated scream> something like that*. *When I’m stressed out*, *I’m pacing when I have that kind of stress*. *So*, *I go out of my house and I walk around the house and speak to the walls*.

### Interventions

Determining what kinds of interventions are desirable requires further research. Preliminary evidence from this study shows that the overwhelming majority (99%) of nurses want higher pay, indicated both directly and by the proportions who would like to return to school to upgrade their qualifications (71% of SNs, n = 17; 48% of ENAs, n = 11). Three nurses employed as SNs and two as ENAs had already gone back to school and were overqualified for the post they held at the time of the interview. They wanted better-paying positions consistent with their qualifications.

Nurses tended to feel overworked. Just under half would like the hospital to hire more nurses because they believe their wards or clinics are understaffed. Many favor hiring more staff to reduce the nurse-to-patient ratio, which, according to nurses, is often higher than it should be due to absenteeism, retirements, or nurses being out of wards for continuing education. ENAs and SNs worry that they are pushed beyond their scope of practice due to staff shortages. One SN (R2I33) who is qualified as an RN said:

*[S]taff nurses and the sisters*, *we are doing the same things*. *Let me ask you*. *If you go through this research*, *they [hospital administration] are going to respond and maybe they say that we staff nurses who are working the same as the sisters [the RNs]*, *they are going to say we are doing the wrong thing*. *I know that sometimes we are doing the wrong thing*. *But there is a shortage of staff and then there is a government not giving us posts*. *But we are forced to do this*.

RNs want more or better equipment (38%) and more transparency about changes from hospital administration (17%). RNs and SNs wanted better management and training to learn to work more cooperatively in their wards and clinics.

Another 40% would like more flexible hours in order to juggle the demands of paid work with child- or elder-care, their own healthcare, family events, long commute times, and safety concerns. ENAs and SNs, especially those with children, want more predictable pay for the overtime that they do, which they said sometimes took months to receive in the existing pay systems.

Several volunteered that counseling could help nurses cope with problems like emotional distress in the hospital, which they take home with them, and anxieties about finances that connect their paid work with their lives outside of it. An RN (R2I35) in her early 30s tied the emotional strain of paid work together to her seemingly-personal difficulties in response to a question about what could improve her life at the hospital or outside of it:

*Don’t ask what I’m doing, ask how I am doing*. *That’s all. Inside. That thing makes a difference to me*. … *Ask how I feel. How do I cope being in the hospital, seeing stuff, you know? Maybe things like rehabilitation*. *We need those kinds of things [support] because we see a lot of things. People are dying in front of you*. *It’s not nice. Emotional. Things have that impact*. *Maybe every month we need rehabilitation or talk about these problems that we have*. *In this institution there are things that could be done to help nurses cope*. *Sometimes they don’t cope*…*you’ll see that a person died in front of you*. *We don’t have counseling for that*. *We need such things*.

*Counseling would be something that would help us deal with these situations*. *We need something like that because not everyone can cope because they see death and think, “I will be the next one or someone from my family” and it’s not nice*. *And you see families crying*. *The work is emotionally demanding*. *If we had weekly or monthly programs for nurses to come to counseling, they could make us stronger*. …

*When you cough out something it relieves you*…*Us nurses also face our own problems and we need someone to sit down and talk about it and get advice, so at least you get relieved*. *But we can’t because we don’t have those people*. *We don’t…we just keep it to ourselves until <trails off and shrugs shoulders>…If we need to cry, all of us, we can cry, just at least the thing that is inside you is out*. …

*What you are doing, wena, ne, what you are doing yourself, if we were at least to have that, someone who will speak to us*. *Maybe one day is one group of people and then another group another day*. *To let us share our concerns and experiences so that we will feel better*. *We each have different problems; I have different problems from her but I might be able to help her and she might be able to help me too*. *Something like that to sit and talk*. …

*I was sitting at the desk and I said*, *“Please interview me also*.*” Do you remember*? *I said “what about me*? *Why didn't you interview me*?*” I wanted an interview also*. *I heard them [nurses the PI interviewed] when they said*, *“Know what*, *this really helped us*. *We just talked*.*” But they didn’t want to say what they were talking about*. *But you could see in their faces to say*, *“Oh…they felt better*.*”* (emphasis in original)

Others, like this SN (R2I22), also described a sense of relief after talking about the challenges they face:

*It’s so important to talk to someone*. *Being open*. *The questions that you’ve asked me, they’ve made me so open*. *And I’m open at all times, but they made me so open that I feel relieved of everything*. *I feel that I don’t even have that financial stress anymore because of talking about it to you*.

Another, also an SN (R2I33), described why talking may be effective:

*Oh, I’m relieved*! *<laughs>*

*Q*: *You’re relieved*? *Why is it a relief*?

*A*: *No*, *I was having a stress with the money*. *Especially this month*.

*Q*: *Did you tear up because you're relieved*?

*A: <tears up again> <laughing> Yoh*! *I’m holding myself. I don't know who can talk to me about this stress. When you talk to somebody, you're not alone*. … *Sometimes you sit and you think about how you are doing, if you can do more work, but you can't have more time because of the work [you already have]*. *Sometimes I get so stressed and I think let me go and pay all that amount that I am owing, it's fine*. *And when you go to buy groceries you can see this month you can only buy the important things*. *Rice, maize meal, and meat only*. *That's it*.

An RN (R1I17) requests financial counselors:

*Maybe after you finish with the interviews maybe then we can bring in some counselors to help people*. *Financial counselors. Because maybe we don’t know how to handle finances all in all*. *Because sometimes you can find someone earning 2,000 or 5,000 rand but she can manage her finances more than a person who earns 20,000*. *But at the end of the day she still has got debts to pay*. *So maybe in a way, after you finish this then you can help*.

*The debts that I have now, because after the OSD [occupation specific dispensation] the salaries were better*. *But we’ve got the long-term problems that we had before that are still having an impact now*. *So, it takes a long time. But maybe if we had some advisor that could advise us financially maybe we can manage our finances better*. *Maybe there are unnecessary things [accounts] that you open, maybe it’s not even necessary*. *Things like that*.

## Discussion

*Q: Tell me a little bit about stress at work and stress at home*.

*A*: *What can I say*? *At work*, *there's not stress like* *bad* *stress*. (emphasis in original)

—An ENA (R1I29), 34, single mother of three, one child living with her and two being raised by her mother in another province.

Nurses share a belief in the conventional wisdom that nursing is a stressful occupation, and therefore they expect stress at the hospital. In contrast, the “bad stress” noted by the nurse quoted above comes from stressors that appear to have origins outside of paid work. For most nurses, those stressors are at least as strong as those inside the hospital. Some even expressed a sense of relief and refuge from the anxieties of their home-lives when they were in the hospital.

High levels of familial dependency are significant sources of stress. Dependency is a result of a number of factors: the macroeconomic environment–in 2018 the expanded unemployment rate, which includes discouraged work-seekers, among black Africans was 37% for men and 45% for women; morbidity and premature death among family members who leave children in need of support; and women’s disproportionate responsibility for children in general, which is a recognized norm across different types of families and other forms of social difference such as race and class in South Africa [40,41]. As of 2017, for over 60% of births, mothers did not even fill in information about their child’s father on the state-mandated birth report [42]. Most children in the country are raised without fathers contributing socially or financially [15,43].

Gender norms regarding children may be especially strong for black women due to apartheid legislation including the Group Areas Act of 1950, the Population Registration Act of 1950, and the Pass Laws Act of 1952. In conjunction with the mining-based economy these laws entrenched the migrant labor system that drew male laborers into urban areas and limited women’s mobility. The norm is a function of “tradition” as well in the sense that black women’s identities are historically attached to marrying, having a well-kept home, and having children [44]. Marriage plays an important role in marking a transition from girlhood to adulthood in South Africa, and married women are held in higher esteem than their unmarried counterparts [12]. Although marriage and fertility rates have both fallen, having children remains a cornerstone of most women’s gender identity, whether means raising them with a partner or not [39,44,45].

Women tend to manage households with greater numbers of children, older people, adult children, and extended family members than men, a well-documented phenomenon in developing countries including in South Africa, where 44 percent of households are female-headed [17,37,46–49]. Likewise, working-age people in female-headed households support more people (0.98) than those in male-headed households (0.60) [17], and female-headed households tend to have fewer income earners than male-headed households [17]. Put simply, women support more dependents *and* their households are more dependent on women’s earnings, both of which likely contribute to women’s sense being under pressure.

But despite the *appearance* of two separable, distinct sets of stressors, one from the hospital and one from the home, nurses’ stress outside of paid work tends to be directly connected to aspects of that work, such as shift scheduling, nurses’ pay, and geographic constraints. The demands of nursing combine with gender norms and the economic realities of dependency described above to contribute to chronic stress among nurses.

The nursing occupation profoundly impacts nurses' lived experiences outside of paid work. For some, nursing determines where they live and who raises their children. Nursing strongly influences the dynamics of their relationships with partners and family members, generating complex interdependencies around financial support, debt, and care-giving. In practice, the stressors of “work” and “home,” or paid work and unpaid reproductive labor, are inextricable. Even the distress experienced when patients die is connected to nurses’ own families. “*We need something like [counseling] because not everyone can cope because they see death and think*, *“I will be the next one or someone from my family” and it’s not nice*. *And you see families crying*.” There are no separable spheres of paid work and home: as the human worker physically moves through time and space, her resources–and her stress–travel with her.

For almost all participants, the shift-work structure of nursing contributed to stress inside and outside of paid work. Many challenges nurses confront entail multiple sources of stress and strategies for addressing challenges can generate new problems. For example, the shift schedule renders childcare practically impossible for a lone single mother, which may cause a nurse to raise her children in a household other than her own.

Child-leaving has a history formed, in part, by the apartheid-era migrant labor system in which some black women were employed as domestic workers in urban areas and their children were raised in a family home in a “bantustan.” Other historical and contextual influences include cultural preferences and the impacts of HIV [36,37]. Their schedules caused nurses to struggle with responsibility for childcare during a 14-hour day (including their average commute). The practice connects nurses firmly to other households, both emotionally and financially, which has emotional and material costs as well as benefits for all involved. Some scholars worry that, “Without their biological mother to defend their interests, children might be seen as a burden, be moved between different households, have their emotional needs neglected, and girls might be exploited as cheap labour in the home” [15].

At a superficial level, a nurse with a child being raised elsewhere may be concerned about the distance; she fears that the child will hurt himself; he might fall in with the wrong crowd. At a deeper level, she may be anxious that she is depriving her child of her presence, that he is suffering because of her job. She may be worried that he will not grow up the way she wants him to. She can feel guilty for being too tired to play with her son the way he would like after she travels for hours in a minibus taxi to visit, as one described her experiences of her one weekend-per-month trips home. During the visit she also feels that she should give her sister a break from taking care of the kids. In other words, the source of her distress is not (merely) that the shift schedule *conflicts* with other demands on the nurse, in this case, providing childcare. Instead, the demands on the nurse–now to travel to see her son who is being raised elsewhere–*exist* in the first place *because* of the shift schedule in gendered context.

Further, the challenge of the shift schedule is not resolved by the nurse leaving her child with her sister, it is “coped with.” The strategy itself can produce a range of other stressors including guilt, anxiety, sadness, insecurity, and self-doubt. In this way the stressors that *appear* to be based in the home are directly connected to, or even originate in, the conditions of nurses’ paid work in the existing social environment.

That distress is also the emotional background from which the nurse steps out of the minibus taxi and into the hospital in the morning. It is not surprising, then, that for some nurses paid work can function as a distraction from the emotionally exhausting demands of the family members that they support. This does not mean, however, that paid work is “less demanding” than unpaid work or that nurses’ paid work-lives are “less stressful” than their lives outside of work. Stresses in nurses’ paid work-lives and in their lives outside of paid work are not separable, therefore they are not comparable in a meaningful way. Rather, nursing-as-distraction suggests that paid work allows the worker to focus on a more immediate set of tasks and the shorter-term aim of helping a patient heal. Nurses are typically busy while they are in the hospital, all the more so because of absenteeism, undoubtedly driven partly by stress [50]. Focusing on a discrete set of tasks that typically require engagement with patients and a significant measure of caution to avoid things like needle sticks, allows nurses to more easily avoid thinking about their stress. In comparison, a more concerted effort is required to avoid thinking about stressors at other times, such as during their commutes, a period when many experienced distress.

The nurses’ emotional responses to being asked, “How are you?” suggests that paid-work-as-distraction allows nurses to develop a fragile veneer of nonchalance that conceals distress. Nurses tend not to talk about their problems with each other, which further enables them to avoid thinking about stressors at work. It allows them to cope with their stress. However, this silence may simultaneously undermine their mental and physical health. It is informed by, and combines with, neoliberal ideology to cause nurses to interpret stressors as individual or household problems; as personal, private struggles that they bear alone.

The purported split between “work stress” that is simply “part of the job” and “home stress” that is a “private concern” can cause nurses to perceive widely-shared problems as personal failures for which they might feel ashamed.

This point is not just theoretical; it is of great practical and material importance: gendered value systems dictate that issues associated with paid work alone are the “proper” terrain for unions and workplaces to attend to, and reinforce the idea that other stressors are private issues. But when a stressor is widely shared among workers in a particular occupation, it suggests the stressor is in fact associated with their paid work. In other words, *it is an occupational concern*.

Traditionally, labor union advocacy is about paid work, not unpaid reproductive labor. Unions mainly advocate around pay and working environment because stress that is “part of the job” is largely non-negotiable. The scope of negotiation may also be linked to the gender of union leadership, which is 70% male across the unions of which the nurses were members [14,51]. One nurse (R2I23), discussing policy-makers and union leadership, said *“[S]ome of the things*, *they can't see them*, *they don't know them*, *they don't know what is happening*.*”* But there are interventions that could help address some of the distress black women nurses experience. A range of potentially beneficial changes—which include higher pay, on the grounds of dependency on women—may help nurses cope with, if not actually ameliorate, interrelated stressors.

Nurses recognize that shift work is a non-negotiable stressor but they want some flexibility in determining their schedules. Having a free or subsidized creche on-site that is available for nurses’ entire shifts could alleviate some of the difficulties of childcare that make the shift schedule such a challenge. More cooperative working environments and inclusive decision-making would be a welcome change for some who noted tensions in their wards, often between lower tier nurses and the RNs above them in the nursing hierarchy. Between their work at home and in the hospital, where nurses believe that understaffing is a problem, they feel overworked. Even if understaffing is not a problem according to legally mandated patient-nurse ratios, the fact that nurses *feel* overworked and become burnt out is reason enough to consider adding staff. Barring significant and rapid changes in gender roles and the distribution of unpaid reproductive labor, raising nurses’ incomes could allow them to pay for help with domestic tasks.

Some nurses want counsellors to speak to about the challenges they confront like debt, relationship difficulties, and psychological distress; and access to resources to answer related questions. In another study a nurse expressed the perceived demand for counseling, “[she] felt counselling was impracticable. She said: ‘If they were to offer us counselling, the whole hospital would be queuing for counselling, because just about all the nurses are under stress’” [14]. The quotations in which nurses refer to the interviews for the present study are indicative of how extreme and how internalized these stressors are. The tears shed in interviews and the relief experienced by some are suggestive of nurses feeling profoundly alone, exhausted, unappreciated, and unsupported.

## Conclusions

Without exception, study participants experienced high levels of stress.

The nursing occupation in South Africa is embedded in a context of extreme racial and gender inequality, very high unemployment, apartheid geography, and social beliefs about gender. Gender norms enable family members’ claims on time and resources that can place an immense amount of pressure on women, whether or not her dependents reside within her household. Using monthly remittances, this study generated a conservative estimate of dependency—five people per nurse—that should be considered a baseline level. The estimate would be much higher if the recipients of less frequent money sending and spending on groceries were included. It would be much, much higher if the beneficiaries of spending on funerals, weddings, and household construction were included.

The hospital and nurses’ household networks are inseparable sources of distress for nurses. The nurse herself is a care worker who links the two. Her distress about, for example childcare or child-leaving, ostensibly a “household” or “private sphere” concern, is generated by her need for 14 hours of childcare per workday between her paid work and her commute, which is a requirement of her work in the hospital. Under the conditions that nurses currently work, the emotional exhaustion that nurses bring to the hospital, seemingly “from home” constitutes a predictable outcome of her paid work as a nurse. That exhaustion is a key aspect of being a nurse in a public hospital in South African society, and one around which occupational health and mental health strategies should be designed.

Nurses are a special kind of worker. They are typically women who do shift work, already a small and overburdened group. And due to historical circumstances that radically circumscribed occupational choice for Africans in South Africa, it is black women who have been funneled into positions that place extraordinary demands on them, with significant consequences for their health and well-being. If that alone were an insufficiently compelling reason to pursue new strategies to support them, nurses are also a valuable resource critical to the functioning of any public health system.

Since nurses’ heavy workload derives partly from gender norms, individual nurses may be limited to finding coping mechanisms to manage their workload-related stress. Therefore, until gender norms change, and there is some evidence that they are changing, unions and hospitals play a critical role in easing this strain by advocating for better working conditions and providing access to mental health resources [45].

Several interventions should be considered, many not conventionally related to the workplace environment, but needed to address the complex lives and communities that nurses inhabit. Modules in nurses’ education about financial planning and budgeting, debt counseling, retirement, and information about buying a home, may proactively give nurses skills to deal with what we found to be major stressors. Resources for financial advising, debt counseling, and legal advising about child support, divorce, and domestic violence should be made available on an ongoing basis. Mental health interventions specifically addressing anxiety and depression may be useful. Relationship counseling could help nurses navigate difficult discussions about the distribution of work in the household. Enabling nurses to gather into support groups may help as well. Simply gaining a sense that an individual nurse is not alone in her struggles would likely improve her wellbeing and may encourage her to seek out further help.

In negotiating pay, unions, politicians, and managers should consider the fact that some nurses are supporting multiple households and up to 14 or 15 people, in part because the demands of nursing render it difficult to maintain one’s family in a single household.

Finally, more needs to be done at a societal level to change the gender norms that systematically put women at a disadvantage, and it must be done in a way that does not provoke violence against women. That domestic violence was not presented as a challenge for nurses themselves may mean that it is truly exceptional among the group or that it is perceived as shameful generally or specifically in the interview context. Alternatively, the lack of mention could also mean that domestic violence, or the threat of domestic violence, is so commonplace as to seem not notable. According to Jewkes et al., lifetime prevalence of domestic violence was 24.6% in South Africa and “widespread tolerance reflects both ideas that the use of violence is often ‘normal’ [and] inevitable, and ideas about gender which legitimate the use of force by men in establishing hierarchical control over women” [39].

Black nurses bear a disproportionate burden when it comes to expectations around relationships, support to extended families, and childcare. In the same way that South Africa is attempting to slowly address the legacy of apartheid and the gross racial misallocation of resources, the country needs to begin pro-actively addressing the related challenges that black women confront within the society.

## Supporting information

S1 Questionnaire (DOCX)

S1 Dataset SA nursing union leadership. (XLSX)
