# Supplementary material for: The integration of occupational- and household-based chronic stress among South African women employed as public hospital nurses
Source: PLoS One. 2020 May 1;15(5):e0231693. doi: 10.1371/journal.pone.0231693 (PMC7194431; doi:10.1371/journal.pone.0231693)
Supplement: S1 Questionnaire — (DOCX) [file pone.0231693.s001.docx]

**Nurses at Charlotte Maxeke Academic Hospital**

Professor Jennifer Cohen

Research Associate, Society, Work, and Development Institute, University of the Witwatersrand &

Assistant Professor of Economics, Whitman College

**Invitation to Participate**

This research project aims to understand the paid and unpaid work that nurses do inside and outside the home. The project may provide information that will help inform policymakers about the daily lives of nursing workers.

You are invited to be interviewed in a process that will take approximately one-to-two hours in a restaurant or other quiet location nearby Charlotte Maxeke Hospital.

**Compensation**

Participants are entitled to a meal and reimbursement for travel costs. (Limit R100.)

**Risks**

There are minimal risks to participating in this study. Participation is voluntary. If you are uncomfortable you have the right to stop the interview at any time without consequence and I will destroy any notes or recordings that I have made.

**Benefits**

Your participation in this project will contribute to policy-oriented research on work, employment, and the household.

**Privacy**

As a participant, your privacy will be protected and your identity will remain confidential. With the exception of the researcher, no one will have access to interview notes, recordings, or any information which could identify you personally.

**Further information**

If you have any questions about your rights as a participant or about the research, please contact me at 084 050 1401 or [jcohen@whitman.edu](mailto:jcohen@whitman.edu)

Consent to be interviewed: _________________________________ Date: _________________

To be filled in at time of interview (optional):

Consent for interview audio to be recorded: __________________________________________

Nursing in Johannesburg - Round 1

Name: ____________________

Study ID: _________________________ (office use only)

Ward: ____________________________

Union: _________________________

DOB (or age): _____________________

Where from: _________________

Position: ________________________

Background:

Matric:

Qualified as _______________ Year: ___________

Currently live in:

Who lives in that house with you:

Kids:

Parents:

Siblings:

HH network - N of HHs:

Marital status:

Married - Partner/long term relationship - Divorced - Widowed - Never married

How do you organize your time? Do you cook and clean every day? Do you do those tasks mainly on weekend days?

Describe average day:

Wake up

Leave house

Commute time cost

etc.

On an average day, how many hours per day do you spend doing ________ for the benefit of yourself and the other members of your household:

Cooking __________

Cleaning __________

Caring for children __________

Doing laundry or mending clothing __________

Caring for adults/elderly ____________

Other non-market production? __________

? __________

? __________

Does anyone else in the household contribute to these tasks? Is this a relative or someone that you pay for their help? Or a relative that you pay?

What do you so in your free time (do you have any)?

Is this job at the hospital your only job? Do you “moonlight” in private hospitals? Do you do any other paid work?

On an average day, how many hours per day do you spend doing paid work?

Would you like to work more hours, fewer hours, or the number of hours you currently work? Why?

Would you say that you work hard? Why or why not?

What are your main reasons for working?

Are you satisfied with your **paid** work? What do you like/dislike about it?

Like:

Dislike:

Are you satisfied with your **unpaid** work? What do you like/dislike about it?

Like:

Dislike:

Are you satisfied with the **balance of paid and unpaid work**? What do you like/dislike about it?

Does your paid or unpaid work cause or aggravate any health problems?

Do you feel anxious?

What are some sources of anxiety? Is your anxiety about work, about the house hold, or both?

Do you think that women face any challenges in paid work that men don’t face?

Likewise, blacks and whites, South Africans and immigrants,

Do you think that women face any challenges in unpaid work that men don’t face?

Do you think that caring for children is primarily women’s responsibility?

Do you think providing income is primarily men’s responsibility?

Do you send income out of HH to support others? In kind transfers? “money, food, or any other kind of contribution from people who do not usually sleep in the same house as you at least four nights per week” Child support payments?

- - - Is this money etc. something you send regularly (eg. every month?)

Have you sent money irregularly in the past year - because of an emergency or hardship?

Send money for school fees for children in your extended family? Or for your own children who do not live in your HH?

Weddings or funerals for your extended family members?

Home building/renovation?

Money to start a business?

Gifts of food?

- - - If so, where are those people living?

Do you receive income from people who don’t live in the HH? “money, food, or any other kind of contribution from people who do not usually sleep in the same house as you aat least four nights per week” child support payments?

- - - is this money etc. something you receive regularly (eg. every month?)
    - If so, where are those people living? (specifically, rural-> urban transfers in kind?)

Receiving government grant? OAP and/or child support grant

Do you have relative in your extended family who receive grants?

Thoughts on social grants? How/do they impact you?

Other sources of income? Agricultural income? Investment income? Rental income?

Income from main job: _______________________ (is this take home or gross?)

Income from interviewee secondary job: ___________________

Income from family member: __________________

Income from family member: __________________

Income from family member: __________________

Income from family member: __________________

Income from grants: ___________________

Total HH income: __________________

Out of paycheck:

What is deducted? Retirement cover? Insurance?

Covers, insurance policies, stovels, burial societies - number of people covered on each one?

Retirement policy - provident fund?

Do you have savings in a bank?

What do you pay on debts to banks?:

How much do you owe to banks in total? (does this include credit cards or just loans)

For what purpose did you borrow this money?

What do you pay on debts to other lenders?

How much do you owe to other lenders in total?

For what purpose did you borrow this money?

Do you pay school fees? How much per month and for how many children?

Do you pay rent? How much per month?

Do you pay a mortgage? How much per month?

Do you own a car? Do you pay for that every month?

What kinds of workspace changes do you think would improve your worklife?

Are there workspace changes that you think would improve your homelife?

Other things to share?

Questions for me?
